# Clinical Application of Mesenchymal Stem Cell-Derived Extracellular Vesicle-Based Therapeutics for Inflammatory Lung Diseases

**DOI:** 10.3390/jcm7100355

**Published:** 2018-10-14

**Authors:** Yu Fujita, Tsukasa Kadota, Jun Araya, Takahiro Ochiya, Kazuyoshi Kuwano

**Affiliations:** 1 Division of Respiratory Diseases, Department of Internal Medicine, Jikei University School of Medicine, Tokyo 105-0003 Japan; tkskdt@gmail.com (T.K.); md986001@yahoo.co.jp (J.A.); kkuwano@jikei.ac.jp (K.K.); 2 Division of Molecular and Cellular Medicine, National Cancer Center Research Institute, Tokyo 104-0045, Japan; tochiya@ncc.go.jp

**Keywords:** extracellular vesicles, microRNA, mesenchymal stem cells, inflammatory lung diseases

## Abstract

It is currently thought that extracellular vesicles (EVs), such as exosomes and microvesicles, play an important autocrine/paracrine role in intercellular communication. EVs package proteins, mRNA and microRNA (miRNA), which have the ability to transfer biological information to recipient cells in the lungs. Depending on their origin, EVs fulfil different functions. EVs derived from mesenchymal stem cells (MSCs) have been found to promote therapeutic activities that are comparable to MSCs themselves. Recent animal model-based studies suggest that MSC-derived EVs have significant potential as a novel alternative to whole-cell therapies. Compared to their parent cells, EVs may have a superior safety profile and can be stored without losing function. It has been observed that MSC-derived EVs suppress pro-inflammatory processes and reduce oxidative stress, pulmonary fibrosis and remodeling in a variety of in vivo inflammatory lung disease models by transferring their components. However, there remain significant challenges to translate this therapy to the clinic. From this view point, we will summarize recent studies on EVs produced by MSCs in preclinical experimental models of inflammatory lung diseases. We will also discuss the most relevant issues in bringing MSC-derived EV-based therapeutics to the clinic for the treatment of inflammatory lung diseases.

## 1. Introduction

Extracellular vesicles (EVs) are naturally secreted from many eukaryotic cells and they have a key function in cell-to-cell communication by transferring their components, such as proteins, microRNA (miRNA), mRNA, long non-coding RNA, lipid mediators and even mitochondria that have biological relevance [1]. Cells release diverse EVs, including exosomes, microvesicles and apoptotic bodies [2]. They are distinguished by specific membrane markers, origin and size (exosomes, 50–150 nm; microvesicles, 100–1000 nm; apoptotic bodies, 50–2000 nm). EVs have been detected in body fluids, such as blood, urine, bile, bronchoalveolar lavage fluids (BALF), breast milk, saliva and feces [3,4]. EVs participate in a variety of normal physiological processes [5,6], as well as pathological processes of various diseases, including cancer [7]. The biological functions of EVs and their components vary according to their cellular origins. While EVs exhibit some common components, they also express molecules that reflect the originating cells. For instance, B cell-derived EVs carry functional major histocompatibility complex (MHC)-peptide complexes on their surface and exhibit T cell stimulatory capacity [5], suggesting that cells utilize their EVs to support their functional roles as cells. The other major interest in the EV research area is the potential therapeutic applications of various EVs. Specifically, EVs that have the potential for repairing and regenerating damaged tissue may have a therapeutic ability for refractory disorders that produce irreversible tissue injuries. 

In the past several decades, the development of stem cell therapy and its continuous advancement has gained immense attention. Among all stem cell types, mesenchymal stem cells (MSCs), which have been intensively studied, hold great promise in disease treatment, especially in the fields of regenerative medicine, including inflammatory lung diseases. MSCs are multipotent cells present in bone marrow (BM), umbilical cord vein (UC), adipose tissue (AD) and lungs and possess the capacity to stimulate the maintenance, growth and survival of other cells. These cells have thus attracted much attention as a cell-based treatment for the regeneration of injured lungs, such as in acute lung injury/acute respiratory distress syndrome (ALI/ARDS), chronic obstructive pulmonary disease (COPD), silicosis and idiopathic pulmonary fibrosis (IPF) [8]. However, cell therapy has several limitations, including invasiveness of cell collection procedures and the multiple doses needed to maintain the therapeutic effect [9,10]. In recent years, studies have shown that MSCs can also achieve a therapeutic effect in vivo via direct differentiation and paracrine action [11,12]. In particular, their paracrine ability, including the secretion of immunomodulatory cytokines, tissue repair-inducing growth factors and small membrane vesicles, are of the most interest among researchers. Remarkably, a rapidly increasing number of reports have suggested that MSC-derived EVs have therapeutic effects in the treatment of several diseases, including kidney injury, myocardial injury, cerebral injury and lung injury. It has been reported that MSC-derived EVs have therapeutic effects for various preclinical models of lung diseases [13,14]. Current challenges with developing EV-based therapies are the lack of standardized approaches to EV isolation and the need for clarification of the pharmacological properties and mechanisms of action of EVs. In this review, we outline the current knowledge of the therapeutic potential of MSC-EVs in inflammatory lung diseases. As new perspectives in this field, we also discuss the challenges underlying the pharmaceutical development of EV-based therapies for inflammatory lung diseases from animal models to clinical development.

## 2. Classes, Biogenesis and Cargos of Extracellular Vesicles

In general, EVs are defined as small membrane vesicles and include exosomes, microvesicles and apoptotic bodies and they are mainly based on their origin of biogenesis. Exosomes and microvesicles comprise the most prominently described classes of EVs. These vesicles are surrounded by a phospholipid membrane and contain cell type-specific components, such as proteins, lipids, RNA and metabolites. Some confusion exists in the literature regarding the term “exosomes” and “microvesicles.” Exosomes are the most well-characterized and widely studied EVs, defined as 50–150 nm-sized derivatives of the endosomal compartment. They are generated from the inward budding of late endocytic compartments, named as multivesicular bodies (MVBs) and are subsequently secreted to the extracellular environment as membranous vesicles upon fusion with the plasma membrane [15]. Exosomes have some evolutionarily conserved proteins, including tetraspanins (CD9, CD63 and CD81), heat shock protein (HSP60, 70 and 90), MHC classes I and II, Alix and Tsg101 [16]. In contrast, microvesicles are formed through the direct budding of the plasma membrane and are in the range of 100–1000 nm in diameter. MV release is MVB-independent and does not require exocytosis. Microvesicles have also commonly been referred to as microparticles and ectosomes. The two classes of EVs seem to function similarly after they are released into the extracellular space [17]. Although the origin of exosomes and microvesicles has been well defined, it is difficult to completely separate or even to discriminate different EV types of similar sizes [2]. 

The intercellular transfer of EV components has been proposed to be a widespread process. The field was massively boosted by the findings of the functional transfer of genetic information in the form of mRNA and miRNA between cells via EVs [18,19,20]. Owing to their stable lipid bilayer membrane structure and their ability to traffic in biological fluids, EVs can transport and transfer bioactive molecules, such as proteins and RNA, between cells. Furthermore, EVs can be released and change their compositions in response to cell activation, such as hypoxia, irradiation, injury and cellular stress [21,22]. Indeed, EVs have been shown to play important roles in a broad range of pathological conditions, including lung diseases, through their cargo [23,24]. EVs derived from respiratory cells and immune cells contribute to the production of various pro-inflammatory mediators, potentially serving as key factors in the lung inflammatory process. 

## 3. Isolation and Characterization of Extracellular Vesicles

**Figure 1:**
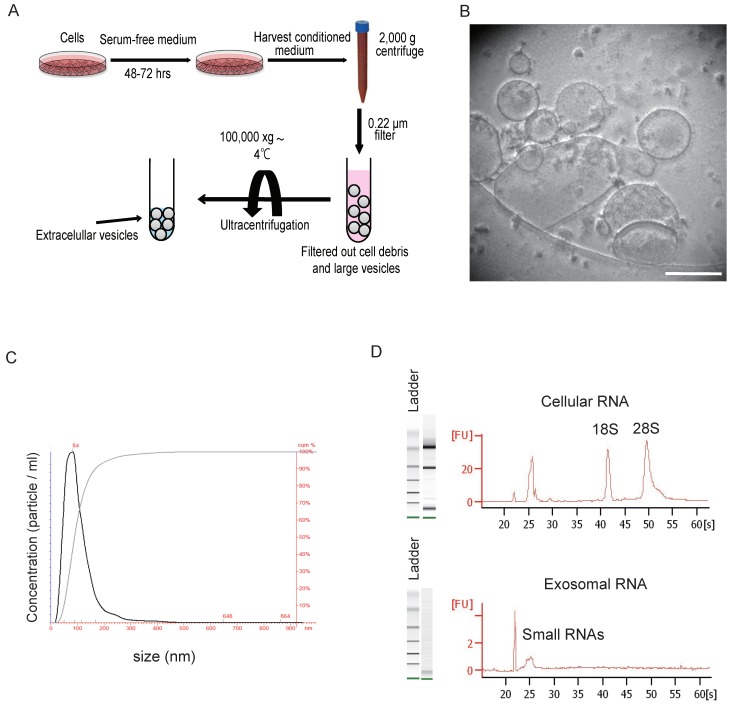
Characterization of EVs isolated by ultracentrifugation. (**A**) Schematic representation of isolating EVs by ultracentrifugation from cultured cells. The method is sufficient to obtain pure EVs for laboratory experiments but it promotes vesicle aggregation and often co-isolate soluble factors and protein. In the clinical setting, it is time-consuming and is not suitable for mass-scale production of EVs. (**B**) Morphology of purified EVs derived from BM-MSCs. Representative phase-contrast transmission electron microscopy images are presented (Scale bar 200 nm). (**C**) Particle size and number determination using NanoSight’s Nanoparticle Analysis instrument (LH10HS) and their proprietary nanoparticle Tracking Analysis software. (**D**) Bioanalyzer analysis of total exosomal RNA by Agilent RNA Pico chip.

The large body of literature describing protocols for EV purification attests to the technical challenges related with this task [25] and the lack of a universally accepted approach. Ultracentrifugation is the most common and conventional method to collect EVs. Commercially available kits such as ExoQuick are a fast and simple kit but it is a relatively crude isolation method resulting in many contaminating soluble proteins [26]. Under the high gravity of ultracentrifuge (over 100,000× *g*), EVs are purified and concentrated from raw material (Figure 1A). The ultracentrifugation method is sufficient to obtain pure EVs for laboratory experiments; however, in the clinical setting, it is time-consuming and is not suitable for mass-scale production of EVs. The ultracentrifugation promotes vesicle aggregation and often co-isolate soluble factors and protein [27]. A density gradient-based isolation, which allows the isolation of vesicles based on buoyant density, provides the highest efficiency for EV purification [26,28]. However, the suitability of this method for a clinical setting is questionable because of difficulties in upscaling and automating such a process [28]. Recently, some emerging techniques, such as size exclusion chromatography, acoustic separation, nanotraps, flow filed flow fractionation, have the potential for isolating EVs from various sample matrices, with each method exploiting a particular biophysical trait of EVs such as their size, density, shape, or surface receptors [29,30]. We need an isolation method that distinguishes each type of EV and can facilitate a large-scale production of EVs.

For characterizing isolated EVs, the morphology and structure of EVs can be visualized under transmission electron microscopy (Figure 1B). We can detect isolated EVs by western blotting or flow cytometry using EV markers, such as tetraspanins, heat shock protein, MHC classes I and II, Alix and Tsg101. Using the NanoSight instrument, measurement of the isolated EVs can be performed to determine particle size and the number of particles (Figure 1C). Total RNA extracted from whole cells using capillary electrophoresis expressed the 18S and 28S ribosomal subunits, whereas EVs contained only small RNAs, such as miRNAs (Figure 1D).

The field currently lacks the ability to completely distinguish each type of EV or to analyze EVs at a single-vesicle level. It is important to keep in mind that different EV profiles might be enriched depending on the isolation method and, even when derived from the same cell types, may differ in their conditions and functional properties. Throughout this review, we use the term EV as a general term for all types of vesicles present in the extracellular space [2]. Furthermore, we mention the vesicle types specifically when the origin of isolated vesicles is known in the referenced studies.

## 4. Mesenchymal Stem Cells and Their Characteristics

MSCs are multipotent stem cells present in mesodermal tissue. MSCs can be isolated from various tissues and organs such as bone marrow (BM), adipose tissue (AD) and blood, including peripheral and umbilical cord blood (UC). Furthermore, MSCs has been identified in the lungs and they are important components of the parenchymal progenitor cell niche and orchestrate organ homeostasis and repair following injury [31]. The subpopulations of MSCs isolated from different tissues have various characteristics. Remarkably, AD-MSCs have several advantages over other MSCs. (i) higher yield of cells per gram of tissue, (ii) higher cell proliferation rate than BM-MSCs and (iii) easier access to the cell source because adipose tissue is often discarded after surgical procedures [32,33]. MSCs have the capacity to self-renew and differentiate into multiple cell lineages, including three major mesodermal lineages: osteoblasts, chondrocytes and adipocytes. Homing and migration are other distinctive functions of MSCs. Previous studies have demonstrated the homing potential of MSCs to the injured sites while also exerting their therapeutic effects on the damaged tissues [34,35]. They can also differentiate into cells from unrelated germline lineages, resist immunosurveillance, home to injured tissue and secrete factors with immunosuppressive, anti-apoptotic and trophic effects [36,37]. Furthermore, MSCs have been shown to possess anti-inflammatory and anti-fibrotic properties due to the secretion of various cytokines and soluble factors, which affects various immune cells and promotes tissue generation [38]. MSCs can interact with alveolar macrophages via cell-to-cell contact and promote their reprogramming via the cyclooxygenase-2 (COX2)-mediated prostaglandin E2 production [39,40]. According to the Mesenchymal and Tissue Stem Cell Committee of the International Society for Cellular Therapy established in 2006, the minimal identifying characteristics for human MSCs are [41]: (i) an MSC must be plastic-adherent when maintained in standard culture conditions; (ii) an MSC must express CD105, CD73 and CD90 and lack expression of CD45, CD34, CD14 or CD11b, CD79a or CD19 and HLA-DR surface molecules; and (iii) an MSC must be able to differentiate into osteoblasts, adipocytes and chondrocytes in vitro.

For years, MSCs have been applied in treating cardiovascular diseases, stroke, spinal cord injury, kidney injury, lung injury and graft-versus-host disease (GvHD) with remarkable achievements. A meta-analysis reported that an evaluation of 36 clinical trials found no association of MSC therapy with significant adverse events, nor malignant transformation [42]. For the MSC treatment to be well tolerated and safe in the short term, however, follow-up of these subjects would be important before conclusions regarding long-term safety could be made. Based on these early studies, the number of clinical trials using MSC therapy has continued to increase. Currently, approximately 40 clinical trials (phase I or II) are specific to investigating MSCs in the treatment of lung diseases [43]. There are published results from 6 completed phase I trials investigating the safety of MSCs for lung diseases, such as COPD [44,45], ALI/ARDS [46,47] and IPF [48,49]. They have reported that there were no serious acute adverse events attributed to MSC therapy in any of the studies. One study showed that patients with COPD and an elevated C-reactive protein (CRP) at baseline showed a significant decrease in CRP levels following MSC treatment, suggesting that MSCs can inhibit the inflammation that is present in COPD [44]. Importantly, two studies showed that no fibrosis or tumor formation was noted on chest CT scans in the MSC-treated groups at either 6 months or 1 year post-MSC treatment [45,49]. Furthermore, one study analyzed lung histology and gene expression changes of the patients treated with MSCs by performing lung volume reduction surgery before and after MSC therapy in COPD patients [45]. By histological analysis of the lung tissues, they found no evidence of induction of fibrotic responses in the lung by MSC treatment. There was neither an increase in α-SMA or pro-fibrotic gene expression, nor in mRNA expression of proliferation markers in the MSC-treated lung tissues. Treatment by MSCs was accompanied by a significantly increased expression of the endothelial cell marker CD31 in the alveolar septa of emphysematous lung tissue, suggesting improved angiogenesis, growth and repair in the injured lungs [45].

Although the clinical trials are progressing, we still lack a complete understanding of the mechanisms underlying the therapeutic properties of MSCs. Some studies on the bio-distribution of MSCs after systemic infusion have indicated that the localization of MSCs at target tissues is rare [50,51,52]. Indeed, it has been reported that <1% of MSCs survive for more than one week after systemic administration [53,54], suggesting that the main effects of MSCs are probably mediated by paracrine mechanisms [55,56]. Many studies have attributed the therapeutic effects of MSCs to their engrafting and differentiation capacity; however, subsequent findings have shown that MSCs likely function through paracrine signaling. The paracrine effects of MSCs are mediated through secretion of a variety of bioactive molecules, including signaling peptides (interleukin (IL)-6, IL-8 and vascular endothelial growth factor), extracellular matrix proteins (collagen and elastin) and others [57]. In 2006, Gnecchi et al. showed that intramyocardial administration of conditioned medium from MSCs could have the same therapeutic effect in reducing infract size as MSC transplantation [58]. Furthermore, Lai et al. showed that MSC mediated the cardioprotective paracrine effect by secreting exosomes [59]. These studies have led to the next stage of MSC research, where MSC-derived EVs have attracted much attention for their potential use in tissue repair and regeneration.

## 5. Functions of Mesenchymal Stem Cell-Derived Extracellular Vesicles

EVs have functions that depend on the phenotype of their parent cell. The potential of MSC-derived EVs to restore and maintain the homeostasis of the tissue microenvironment depends on the biochemical capacity of the protein and RNA transfer [60]. MSC-derived EVs express MSC phenotypic markers, such as CD29, CD73, CD44 and CD105 and can be identified through conventional flow cytometry [61]. Furthermore, characterization of the compositions of MSC-derived EVs has identified several proteins, among which are mediators controlling self-renewal and differentiation. Kim et al. investigated proteomic profiles of EVs derived from human MSCs [62]. They revealed a number of unique proteins, such as surface receptors (PDGFRB, EGFR and PLAUR), signaling molecules (RRAS/NRAS, MAPK1, GNA13/GNG12, CDC42 and VAV2), cell adhesion molecules (FN1, EZR, IQGAP1, CD47, integrins and LGALS1/LGALS3) and MSC-associated antigens (CD9, CD63, CD81, CD109, CD151, CD248 and CD276) [62], supporting a possible role for such vesicles in tissue repair. On the other hand, MSC-derived EVs are enriched by distinct classes of RNAs that could be transferred to target cells and translated into protein, resulting in an alteration of the target cell phenotype [63]. Through mass spectrometry and array analysis, more than 850 unique gene products and more than 150 miRNAs have been identified in the cargo of MSC-derived EVs [64,65]. The miRNAs in MSC-derived EVs are usually related to development, cell survival, differentiation and regulation of the immune system [66]. In addition, MSC-derived EVs contain transcripts involved in the control of transcription (transcription factor CP2, clock homologue), cell proliferation (retinoblastoma-like 1, small ubiquitin-related modifier 1) and immune regulation (IL-1 receptor antagonist) [63]. 

MSC-EVs have shown encouraging therapeutic effects in several different types of diseases, including kidney injury, cardiac injury, brain injury and lung injury [67,68]. The therapeutic capacity of MSC-EVs derived from different organs have been tested in various disease models, demonstrating a similar or even superior functional potential to MSCs themselves [69,70,71,72]. The administration of EVs can avoid many safety concerns caused by MSC transplantation, such as arrhythmia [73], tumorigenesis, ossification, or calcification in tissues [74]. Furthermore, MSCs may lodge and initially obstruct small vessels in organs [50]. EVs, in contrast, have no vascular obstructive effect or apparent adverse effects. These properties suggest that they could be safely and easily used in lung disease therapies.

## 6. Mesenchymal Stem Cell-Derived Extracellular Vesicle-Based Therapeutics for Inflammatory Lung Diseases

MSC-derived EVs have been investigated in experimental models of inflammatory lung diseases, including ALI/ARDS, IPF, silicosis, COPD, asthma, pneumonia, pulmonary artery hypertension (PAH) and bronchopulmonary dysplasia (BPD) (Table 1). Information has emerged regarding the roles of specific miRNAs and other EV components as mediators of the protective effects of MSC administration in preclinical lung disease models but much remains unknown. Currently, the role of EVs in the treatment of lung diseases is an area of active preclinical study.

ALI/ARDS is a common form of hypoxemic respiratory failure in critically ill patients that has a mortality of 25–40% [75]. Severe inflammatory responses cause collateral damage in lung tissue irrespective of the initial cause and patients with ALI/ARDS have high levels of inflammation and circulating cytokines. Unfortunately, clinical trials using anti-inflammatory agents such as glucocorticoids to treat ALI/ARDS have failed to improve outcomes. Currently, MSCs are increasingly recognized as a promising candidate therapy for ALI/ARDS. Regarding MSC-derived EVs for preclinical models of ALI/ARDS, Zhu et al. demonstrated a significant beneficial effect from the intratracheal administration of human BM-MSC-derived microvesicles in an *E. coli* endotoxin-induced ALI mouse model, in part, through the expression of keratinocyte growth factor (KGF) mRNA in the injured alveolus [76]. Human BM-MSC-derived microvesicles reduced lung inflammation and protein permeability, which prevent the formation of pulmonary edema, as measured by the extravascular lung water. The microvesicles also reduced neutrophil infiltration and macrophage inflammatory protein-2 levels in BALF, indicating a reduction in inflammation. Recently, Morrison et al. reported that human BM-MSCs promote an anti-inflammatory and highly phagocytic macrophage phenotype through EV-mediated mitochondrial transfer in the inflammatory environment of ARDS [77]. Human BM-MSC-induced changes in macrophage phenotypes depend on the enhancement of macrophage oxidative phosphorylation. Furthermore, the authors suggested that the changes in alveolar macrophages induced by BM-MSC-derived EVs are sufficient to elicit protection in lung injury in vivo. Monsel et al. reported that microvesicles derived from human BM-MSCs improved survival in ALI from *E. coli* pneumonia via a mechanism partially dependent on KGF secretion [78]. This was associated with enhanced phagocytosis of bacteria by monocytes, with a reduction in inflammation and increased ATP levels in alveolar epithelial type 2 cells. Furthermore, TLR3 agonist pretreatment of MSCs further increased the effects of human BM-MSC-derived microvesicles on monocyte immunoregulatory and phagocytosis properties [78]. Based on these data, EVs released by MSCs were shown to be effective in inflammatory injuries, such as endotoxin-induced ALI and infectious models of ALI.

IPF is a chronic, progressive and irreversible respiratory disease characterized by diffuse alveolar epithelial cell injury and structural remodeling. There is typically no response to general anti-inflammatory therapies such as glucocorticosteroids and immunosuppressants. Some anti-fibrotic agents and adenosine receptor antagonist-based solutions have shown some limited promise. Recently, the administration of MSCs has been used clinically in IPF in a phase I trial [79]. Shentu et al. have shown that human BM-MSC-derived EVs can block TGFβ1-induced myofibroblastic differentiation [80]. Human BM-MSC-derived EVs enter into fibroblasts and may utilize a Thy-1-integrin interaction-dependent pathway to facilitate cell-cell communication by EVs and delivery of EV components. The EVs are enriched for several miRNAs, including miR-630, which targets the pro-fibrotic genes that are upregulated in IPF fibroblasts. They also reported that administration of human MSC-derived EVs at day 14 in mice with pulmonary fibrosis induced by bleomycin significantly downregulated α-smooth muscle actin expression and decreased histopathological fibrosis, indicating the therapeutic effects of these vesicles on the established lung fibrosis through modification of the myofibroblastic phenotype [81].

Silicosis is an occupational lung disease caused by the inhalation of silica particles leading to extensive lung fibrosis and respiratory failure. At present, no effective treatment methods for silicosis have been identified. It has been reported that microvesicles derived from human BM-MSCs effectively reduced the recruitment of inflammatory cells into airways and reduced collagen deposition in lung parenchymal in a silica-induced lung fibrosis mouse model [72]. Although the authors showed that the therapeutic effect of microvesicle treatment was less than that of MSC treatment, further validation may be needed because the vesicles were isolated by using ExoQuick, leading to the isolation of non-exosomal particles [82]. Phinney et al. reported that human BM-MSCs manage intracellular oxidative stress by targeting depolarized mitochondria to the plasma membrane via arrestin domain-containing protein 1-mediated microvesicles. In addition, these vesicles are engulfed and reutilized by macrophages and MSCs simultaneously shed miRNA-containing exosomes that inhibit macrophage activation by suppressing Toll-like receptor signaling (MyD88-dependent), thereby desensitizing macrophages to the ingested mitochondria [83]. Furthermore, Bandeira et al. reported that mouse AD-MSCs and their EVs ameliorated pulmonary fibrosis and inflammation in a late-stage model of silicosis [84]. Interestingly, EV treatment at the higher concentration yielded outcomes comparable with those observed by MSC treatment in this study, promoting enhanced impacts on lung mechanics and macrophage infiltration [84].

COPD is an irreversible inflammatory disease that can display many phenotypes. Little is known about how the multiple structural cells communicate with each other in the pathogenesis of this disease or how they could drive chronic systemic inflammation. Only symptomatic treatment is available for COPD. MSCs have been studied extensively in animal models of COPD due to their tissue-regenerative and immunomodulatory properties. Preclinical studies suggest that cell therapy using MSCs is a potential new treatment for COPD [85]. Kim et al. reported the potential efficacy of human AD-MSC-derived artificial nanovesicles via a FGF2-dependent pathway [86]. They produced AD-MSC-derived artificial nanovesicles, which expressed similar AD-MSC surface markers and growth factors, especially FGF2, compared with AD-MSC-derived natural exosomes. FGF2 is important in lung development and has regenerative capacity. A smaller amount of AD-MSC-derived artificial nanovesicles induced the proliferation of alveolar epithelial cells compared with AD-MSC-derived natural exosomes. Furthermore, the artificial nanovesicles had regenerative effects similar to AD-MSCs and AD-MSC-derived exosomes at lower doses in an elastase-induced emphysema model. These results suggest that artificial nanovesicles may have economic advantages and be clinically applicable to emphysema patients. 

Asthma is a chronic allergic inflammatory disease that can be difficult to treat due to its complex pathophysiology. Asthma involves both large and small conducting airways and is characterized by a combination of inflammation and structural remodeling [87]. Even though several therapeutic strategies are currently available to reduce airway inflammation, no treatment has so far been able to hasten repair of the damaged lungs [13]. Cruz et al. showed that the systemic administration of conditioned medium and EVs from mouse or human BM-MSCs ameliorates *Aspergillus* hyphal extract-induced allergic airway hyperresponsiveness, lung inflammation and the antigen-specific CD4^+^ T cell Th2/Th17 phenotype in immunocompetent mice [88]. In this study, the conditioned medium and EVs were as potent as the MSCs themselves in mitigating the phenotypes. Notably, both the conditioned medium and EVs from human BM-MSCs were generally more potent than those from mouse BM-MSCs in most of the outcome measures. The cross-linking agent 1-ethyl-3-[3-dimethylaminopropyl] carbodiimide hydrochloride was found to inhibit release of both soluble factors and EVs and fully abolished the effects of systemically administered human BM-MSCs but only partly inhibited the ameliorating effects of mouse BM-MSCs. Although the biological differences between human and mouse BM-MSC-derived EVs need to be clarified, these results demonstrated the potent xenogeneic effects of conditioned medium and EVs in an immunocompetent mouse model of allergic airway inflammation. Recently, the same group demonstrated that administration of human AD-MSCs or their EVs had beneficial effects on lung mechanics and inflammation in a model of ovalbumin-induced allergic asthma [89]. Human AD-MSCs and EVs effectively reduced inflammatory processes (reduction in total cell counts and eosinophil percentage in BALF, IL-5 levels in lung tissue and percentage of CD3^+^CD4^+^ T cells in the thymus) and modulated airway remodeling (decreased collagen fiber deposition in the lung parenchyma and airways, reduced TGF-β levels in lung tissues). While the effects of AD-MSCs or their EVs were largely similar, their effects on T cells differed in lung and thymus. The authors proposed that EVs may hold promise for asthma; however, further studies are required to elucidate the different mechanisms of action of AD-MSCs versus their EVs.

PAH is a progressive chronic disease with a high mortality rate characterized by hyperplasia and hypertrophy of smooth muscle cells in small pulmonary arteries and is related to an increase in endothelial cell proliferation that leads to vessel remodeling and consequently, pulmonary hypertension. Despite significant progress in elucidating the molecular mechanisms of PAH and its treatment, PAH is still refractory to most conventional pharmacological therapies. Many studies support an important cytoprotective, anti-inflammatory role for MSCs, with demonstrated efficiency against PAH in animal models. Lee et al. found that murine MSC-derived exosomes prevent the activation of the hypoxic signaling that underlies pulmonary inflammation and the development of hypoxia-induced PAH in a mouse model [90]. The MSC-derived exosomes inhibited vascular remodeling and consequent pulmonary hypertension through suppression of the hypoxic activation of STAT3 and upregulation of the miR-17 superfamily of miRNA clusters, whereas it increased lung levels of miR-204, a known key miRNA that is decreased in human pulmonary hypertension. Recently, it has been reported that rat BM-MSC-derived microvesicle treatment could attenuate the mean pulmonary artery pressure and mean right ventricle pressure and reduce right ventricle hypertrophy and pulmonary remodeling in a monocrotaline-induced PAH rat model [91]. The results showed that intravenous injection of MSC-derived microvesicles or MSCs produces similar beneficial effects for treating PAH. Aliotta et al. also showed that EVs derived from murine or human BM-MSCs can not only prevent the development of monocrotaline-induced PAH but also reverse the pulmonary hypertensive changes, including right ventricle hypertrophy and pulmonary vascular remodeling seen in mice with established monocrotaline-induced PAH [92]. They showed that MSC-derived exosomal miRNAs had increased levels of anti-inflammatory and anti-proliferative miRNAs, including miR-34a, −122, −124 and −127, suggesting that the EVs might modulate pulmonary hypertensive effects based on their miRNA cargo. BPD is a chronic lung disease seen in premature infants who required mechanical ventilation and oxygen therapy for acute respiratory distress. MSC treatment was shown to be effective in the experimental models of BPD [93,94]. Willis et al. reported that human MSC-derived exosomes ameliorated hyperoxia-associated inflammation ad altered the hyperoxic lung transcriptome, resulting in alleviation of hyperoxia-induced BPD [95]. MSC-derived exosomes modulated the macrophage phenotype fulcrum, suppressing the proinflammatory M1 state and augmenting an anti-inflammatory M2-like state. Furthermore, Chaubey et al. showed that human UC-MSC-derived exosomes alleviated hyperoxia-induced BPD and its associated pathologies partially via exosomal tumor necrosis factor alpha-stimulated gene-6 (TSG-6) [96].

## 7. Pharmaceutical Development of Extracellular Vesicle-Based Therapeutics for Inflammatory Lung Diseases

**Figure 2:**
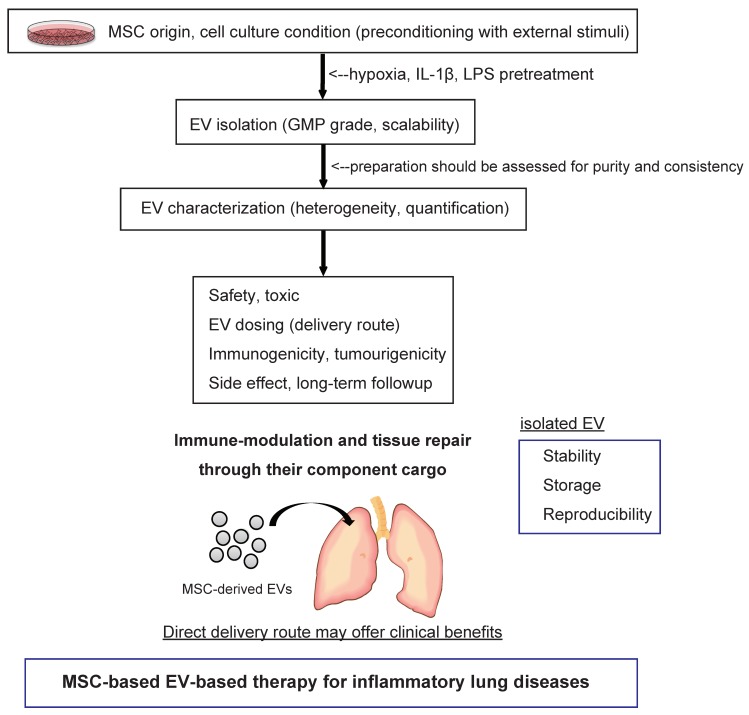
Schematic representation of the proposed therapeutic strategy of MSC-derived EVs in inflammatory lung diseases.

With such promising preclinical findings in various types of disease models, investigators are now tasked with developing safe, feasible and reproducible MSC-EV-based therapies. To date, several clinical applications of MSC-derived EVs have been reported [98]. A published study demonstrated that increasing dosages of MSC-derived exosomes in a patient with severe therapy-refractory acute GvHD, affecting the kin and intestinal tract, was well tolerated and led to a significant and sustainable improvement of symptoms, which remained stable for five months [99]. Globally, at least one clinical trial of MSC-derived exosomes for the improvement of β-cell mass in type 1 diabetes patients has been reported (https://clinicaltrials.gov/ct2/show/NCT02138331?term=MSC+exosomes&draw=1&rank=1). More studies of MSC-derived EV-based therapeutics will be initiated in the near future (Table 2). However, there remain significant challenges to translating this therapy into the clinic. We briefly summarize the most relevant issues to be addressed from various perspectives, such as EV quantification, production, storability, delivery route and potential side effects [100] (Figure 2).

First, it has been reported that the compositions of EVs reflect the cell culture conditions and microenvironmental stimuli that triggered their release. Indeed, the effects of MSC-derived EVs can be potentiated under specific conditions, such as hypoxia [101] and stimulation with growth factors [97,102], which not only increase EV production but may also modulate their components, leading to an enhancement of beneficial effects. Song et al. demonstrated that IL-1β pretreatment effectively enhanced the immunomodulatory properties of MSCs, partially through exosome-mediated transfer of miR-146a [97]. They elucidated that the involved mechanism that upregulates miR-146a in MSCs is via IL-1β stimulation, which is packaged into their exosomes and transferred to recipient macrophages where miR-146a regulates M1-M2 transition and finally contributes to the reduced inflammation and increased survival in septic/ARDS mice. These results suggest that IL-1β pretreatment can be a useful strategy to enhance the immunomodulatory ability of MSCs in cases of excessive inflammation. Recently, Ti et al. showed that LPS-pretreated MSCs release more exosomes and contained higher levels of *let-7b*, which contributed to the improved effects of MSCs on wound healing [102]. These studies suggest that preconditioning MSCs with different external stimuli may improve EV production and considerably enhance their therapeutic activity.

Second, EV quantification is essential to understand the basic biological relationships between EVs and their parent cells and hence the underlying interpretation of EV signals. When in vitro functional studies are performed with isolated EVs, the quantitative analysis of the dose-function relationship should be presented [103]. Currently, researchers use several different methods to quantify EV dosage, making the studies difficult to compare to one another. There are a variety of techniques available that are currently used for EV quantification, such as protein concentration and nanoparticle tracking analysis (NTA), tunable resistive pulse sensing (TRPS), flow cytometry and electron microscopy, with each method harboring its own advantages and limitations [104]. Therefore, to help inter-study comparison, we need multiple quantifications using various quantification tools. Among them, normalization based on EV number provides the most direct comparison and represents the most generalizable option. 

Third, scaling up EV production to meet the needs of clinical studies is crucially important. In 2005, Navabi et al. presented the development of a method for the preparation and characterization of good manufacturing practice (GMP)-grade exosomes from the ascites fluid of ovarian cancer patients [105]. Currently, Pachler et al. provided a GMP-grade standard protocol for exclusively human MSC-derived EVs [106]. Additionally, Watson et al. showed that hollow-fiber bioreactors promote enhanced EV production (40-fold greater EVs/mL) when compared to conventional 2D tissue culture EV preparations [107]. It has been estimated that hundreds of micrograms to a milligram of EVs may be needed to treat patients in clinical trials [98]. EV preparation should be assessed for purity and consistency, with all biological materials used in the EV harvesting adhering to the required regulatory compliance. MSC culture should be carried out under strict laboratory conditions to minimize contamination and increase consistency. 

Fourth, the storability of EVs is an important aspect, both for basic research and for clinical applications. Currently, no standardized procedure is available for the storage of EVs. Fresh preparations can be recommended for clinical therapeutics; however, it remains impractical to always use fresh EV preparations. Efforts for optimizing EV storage protocols are being developing for biobanking. Practically, EVs are stored in isotonic buffers to prevent pH shifts during storage and freeze-thaw cycles. Additionally, storage vials can also affect the quality of EVs, as EVs might unexpectedly and irreversibly bind to certain materials [100]. EVs can be stored at −80 °C, in comparison to −190 °C or at 4 °C and remain biological active [100,108]. With a lack of data addressing the impact of storage times and regents on MSC-derived EV stability and efficacy, tailor-made protocols for MSC-derived EVs need to be developed.

Next, the route of administration still requires further clarification. In general, the delivery of EVs for inflammatory lung diseases can be achieved through intravenous or intratracheal injection. Lai et al. showed that bioluminescence and fluorescence-mediated tomography imaging in mice displayed a predominant localization of intravenously administered EVs to spleen, liver, lung and kidney, with detection also possible in brain, heart and muscle within 30 min of injection, before spiking in the urine at 60 min post-delivery [109]. On the other hand, there are no reports regarding the biodistribution of EVs by intratracheal injection in vivo. In general, intratracheal injection of biological therapeutics for clinical use has some primary advantages over systemic delivery [110]. The direct route may offer clinical benefits, including a lower requirement of EV dose and the reduction of undesirable systemic side effects. The delivery allows direct access to lung epithelial cells, which are important cell types in a variety of pulmonary disorders. A better understanding of the relationships between delivery route, dosage and biodistribution is required to ensure therapeutic biosafety.

Finally, the functions of EVs in mediating horizontal genetic composition transfer raise the potential of risks associated with the uncontrolled transfer of genetic information between cells. We propose that patients who receive MSC-EVs be closely monitored for several years for the theoretical risk of immunological responses and tumor formation. Furthermore, to avoid unwanted immunological events and monitor off-target effects, we should monitor the immunogenicity of EV-based therapies. It is also crucial to investigate the optimal dosage of MSC-EVs and the effect of their repeated administration to achieve the best therapeutic efficiency while minimizing undesired toxicity and serious side effects.

## 8. Conclusions

In this review, we have summarized the current development and recent knowledge of MSC-derived EV-based therapeutics. MSCs have drawn much interest for their therapeutic effects in immune modulation and tissue remodeling. However, the mechanisms by which MSCs might mitigate inflammation and injury are not completely understood and likely involve multiple pathways mediated by the release of soluble factors and EVs. MSC-derived EVs demonstrate several possible advantages over cell-based therapies in terms of their use in regenerative medicine. There is a growing body of evidence indicating that MSC-derived EV-based therapies for inflammatory lung diseases are evolving to become viable treatment options for clinical application. Various studies indicate that MSC-derived EVs exert their effects via the horizontal transfer of proteins, mRNAs and miRNAs. MSC-derived EVs have therapeutic potential for various types of lung inflammatory conditions because of the heterogeneity of EVs. We must eventually clarify the molecular mechanisms of the EV components at the single-vesicle level, leading to the establishment of targeted therapies for specific disease conditions. Several problems need to be addressed before the clinical use of EVs can become widespread. It remains a challenge to develop platforms for the production, storage and handling of clinical grade EVs in a reliable and reproducibly quantifiable manner. MSC-derived EV-based pharmaceuticals and subsequent clinical trials demand the resolution of several technological and mechanistic issues and further investigations in the field of inflammatory lung diseases could provide strong evidence for bringing MSC-derived EV-based therapies to the clinic.

## Figures and Tables

**Table 1:** Application of MSC-derived EVs in preclinical models of inflammatory lung diseases.

Experimental Model	EV Source	EV Delivery	Mechanisms/Target Cells	EV Dose	EV Isolation	Reference
ARDS (*E. coli* endotoxin)	Human BM-MSCs	IT/IV	KGF-expressing EV transfer	EVs released by 3 × 10^6^ MSCs over 48 h	UCF	[76]
ARDS (*E. coli* endotoxin)	Human BM-MSCs	*ex vivo*	EV-mediated mitochondrial transfer	EVs released by 15 × 10^6^ MSCs over 48 h	UCF	[77]
ARDS (caecal ligation and puncture)	Human UC-MSCs	IV	Exosomal miR-146a transfer to macrophages	30 μg protein	UCF	[97]
Pneumonia/ALI (*E. coli* pneumonia)	Human BM-MSCs	IT/IV	KGF-expressing EV transfer	IT; 3–6 × 10^6^ MSCs over 48 h/IV; 9 × 10^6^ MSCs over 48 h	UCF	[78]
IPF (bleomycin)	Human BM-MSCs	IV	Thy-1-expressing EV transfer to fibroblasts	50 μg protein	UCF	[81]
Silicosis	Human BM-MSCs	IV	not reported	10 μg protein	ExoQuick	[72]
Silicosis	Mouse or human BM-MSCs	IV	EVs to outsource mitophagy and shuttle miRNAs	40 μg protein (−3 × 10^11^ EVs)	UCF	[83]
Silicosis	Mouse AD-MSCs	IT	not reported	EVs released by 1 × 10^6^ MSCs over 24 h	UCF	[84]
COPD (elastase)	Human AD-MSCs	IT	EV transfer to epithelium (FGF2 signaling)	EVs released by 1 × 10^5^ MSCs	UCF	[86]
Asthma (*Aspergillus* extract hyphae)	Mouse or human BM-MSCs	IV	not reported	EVs released by 3 × 10^6^ MSCs	UCF	[88]
Asthma (ovalbumin)	Human AD-MSCs	IV	not reported	37 μg protein	UCF	[89]
PAH (hypoxia)	Mouse BM-MSCs human UC-MSCs	IV	EV transfer to endothelial cells suppress STAT3 signaling	10 μg protein	UCF	[90]
Rat PAH (monocrotaline)	Rat BM-MSCs	IV	not reported	30 μg protein	UCF	[91]
PAH	Mouse or human BM-MSCs	IV	EV miRNA transfer	25 μg protein	UCF	[92]
BPD (hyperoxia)	Human UC- or BM-MSCs	IV	EVs modulate the macrophage phenotype	0.9–3 μg protein	UCF (OptiPrep)	[95]
BPD (hyperoxia)	Human UC-MSCs	IP	TSG-6-expressing EV transfer	2.4–2.8 μg protein	UCF	[96]

ARDS: acute respiratory distress syndrome, ALI: acute lung injury, IPF: idiopathic pulmonary fibrosis, COPD: chronic obstructive pulmonary disease, PAH: pulmonary artery hypertension, BPD: bronchopulmonary dysplasia, BM: bone marrow, UC: umbilical cord, AD: adipose tissue, MSC: mesenchymal stem cell, IT: intratracheal, IV: intravenous, IP: intraperitoneal, KGF: keratinocyte growth factor, TSG-6: tumor necrosis factor alpha-stimulated gene-6, UCF: ultracentrifugation.

**Table 2:** Current and past NIH registered clinical trials investigating MSC-derived EV-based therapeutics.

Disease (Number)	Clinical Trial Phase	EV Source	EV Delivery	EV Dose	EV Isolation	Reference
Type 1 diabetes (*n* = 20)	Clinical trial Phase 1, open label	UC-MSCs (allogeneic)	IV	EVs released from (1.22–1.51) × 10^6^ cells/kg, day0 and day7	not reported	NCT02138331
Macular holes (*n* = 44)	Clinical trial early Phase 1	UC-MSCs	dripped into vitreous cavity	50 or 20 μg/10 μL PBS	UCF	NCT03437759
Acute ischemic stroke (*n* = 5)	Clinical trial Phase 1,2, open label	MSCs (allogeneic)	stereotaxic injection	200 μg protein, one month after attack	not reported	NCT03384433

UC: umbilical cord, MSC: mesenchymal stem cell, IV: intravenous, UCF: ultracentrifugation.
